# Hematopoietic Progenitors and the Bone Marrow Niche Shape the Inflammatory Response and Contribute to Chronic Disease

**DOI:** 10.3390/ijms23042234

**Published:** 2022-02-17

**Authors:** Yangsong Xu, Andrew J. Murphy, Andrew J. Fleetwood

**Affiliations:** 1 Haematopoiesis and Leukocyte Biology Laboratory, Baker Heart and Diabetes Institute, Melbourne, VIC 3004, Australia; yangsong.xu@baker.edu.au (Y.X.); andrew.murphy@baker.edu.au (A.J.M.); 2 Baker Department of Cardiometabolic Health, University of Melbourne, Melbourne, VIC 3010, Australia

**Keywords:** hematopoiesis, bone marrow niche, hematopoietic stem and progenitor cells, myelopoiesis, diabetes, obesity, trained immunity

## Abstract

It is now well understood that the bone marrow (BM) compartment can sense systemic inflammatory signals and adapt through increased proliferation and lineage skewing. These coordinated and dynamic alterations in responding hematopoietic stem and progenitor cells (HSPCs), as well as in cells of the bone marrow niche, are increasingly viewed as key contributors to the inflammatory response. Growth factors, cytokines, metabolites, microbial products, and other signals can cause dysregulation across the entire hematopoietic hierarchy, leading to lineage-skewing and even long-term functional adaptations in bone marrow progenitor cells. These alterations may play a central role in the chronicity of disease as well as the links between many common chronic disorders. The possible existence of a form of “memory” in bone marrow progenitor cells is thought to contribute to innate immune responses via the generation of trained immunity (also called innate immune memory). These findings highlight how hematopoietic progenitors dynamically adapt to meet the demand for innate immune cells and how this adaptive response may be beneficial or detrimental depending on the context. In this review, we will discuss the role of bone marrow progenitor cells and their microenvironment in shaping the scope and scale of the immune response in health and disease.

## 1. Introduction

The continuous production of blood cells throughout the lifetime of an organism demands a tightly regulated, yet highly adaptable, system. Under physiological conditions, the balance between self-renewal and differentiation needs to be strictly controlled to ensure the appropriate production of mature blood cells. This precise control makes certain that the flux into each lineage is maintained and that the relative numbers of mature erythroid, myeloid, and lymphoid cells, that vary significantly, are generated. In the classical view, hematopoietic stem cells (HSCs) reside at the top of the hierarchy and are defined by their self-renewal and multilineage differentiation abilities [1,2]. HSCs are rare (~3,000 to 10,000 per adult human) and mostly quiescent (dividing once every 3 months to 3 years) [3], with the bulk of steady-state hematopoiesis being supported by downstream multipotent progenitors (MPPs) [4,5,6]. Together, HSCs and MPPs are defined as HSPCs. MPPs have a limited self-renewal capability and exhibit a more restricted lineage differentiation potential [1,7]. This highly compartmentalized and structured model of hematopoiesis has been challenged by recent evidence, suggesting that HSCs are not a homogenous pool but are instead a heterogenous mix of cells with distinct behaviors, lineage biases and engraftment potential [8,9,10,11]. Moreover, inflammatory or infectious signals can alter normal HSC fate, lineage output and function, adding to the regulatory complexity [12,13,14,15]. It is now appreciated that the hematopoietic system and its ability to integrate a wide variety of signals ensure that it acts as a key determinant of the host response and a major player in the context of chronic disease.

### Advances in Our Understanding of Hematopoiesis and the Role of the Bone Marrow Niche 

Models of hematopoiesis and HSC lineage commitment are constantly being updated and redrawn as we learn more about the complexity of HSC biology. The classical tree-like model of hematopoiesis depicts long-term (LT)-HSCs at the apex as a largely quiescent population with only a minor role in the homeostatic production of blood leukocytes [4]. LT-HSCs can permanently reconstitute blood when transferred into irradiated hosts [16,17] and differentiate into the transcriptionally distinct short-term (ST)-HSCs, which have a more transient and less durable reconstitution ability [18,19,20]. ST-HSCs give rise to MPPs, which have a negligible self-renewal capacity but are thought to sustain hematopoiesis in the steady state by giving rise to lineage-restricted progenitors, such as common myeloid progenitors (CMPs), granulocyte–monocyte progenitors (GMPs) and others [4,21]. Recent technological advancements have challenged aspects of this traditional model by highlighting the heterogeneity within HSPCs (reviewed in) [8,22]. For example, the MPP pool comprises three subsets each with a unique lineage bias. Based on the surface expression of CD150 and Flt3, MPP2 (erythro-megakaryocytic biased), MPP3 (myeloid biased) and MPP4 (lymphoid biased) have been identified [7]. Under normal conditions, the most numerous MPP4 subset is thought to dominate the hematopoietic output [7,23]. Single-cell transplantation studies have identified a wide range of self-renewal and reconstitution abilities across individual HSCs [9,22,24]. HSCs are now best understood as a heterogenous population of cells with distinct self-renewal abilities as well as potentially having inherent “biases” toward certain blood cell lineages [1,5,8,25]. Rather than a series of differentiation steps through discrete progenitor populations, a view of hematopoiesis as a continuum of lineage commitment stages is now emerging.

HSCs reside within the hematopoietic microenvironment of the BM, called the niche. The concept of the “niche” was first proposed in 1978 [26] and refers to the multicellular network of cells and extracellular matrix components that together support, protect, and direct HSCs and their progeny. These cell types include fibroblasts, endothelial cells, osteoblasts, adipocytes, and chondrocytes, in addition to HSC progeny, such as megakaryocytes and macrophages [27,28,29]. The BM niche is innervated and highly vascularized to supply nutrients and oxygen to proliferating hematopoietic cells but also to the many non-hematopoietic cell types that make up the broader niche ecosystem [30]. Being highly vascularized means that BM progenitor populations and soluble factors can be released into the circulation enabling communication between the BM and peripheral tissues. In this sense, the BM compartment acts as a critical endocrine and immune organ. As we will cover later in the article, single-cell sequencing approaches have enabled a deeper exploration of the network of cells that comprise the BM niche and have led to a more detailed understanding of their transcriptional programs [31,32,33]. Communication between HSCs and the BM niche occurs via the production of paracrine factors and direct physical interactions (e.g., between HSCs and extracellular proteins or cells of the niche). This complex network of signals combines to preserve HSCs and their self-renewing abilities with some evidence that distinct zones within the BM niche may exist for certain HSC subpopulations [34,35,36]. The interruption or dysregulation of these interactions by infection, chronic disease or ageing is sufficient to disrupt HSC behavior and alter hematopoietic outcomes [37,38,39]. The importance of the BM niche in coordinating hematopoiesis and how the breakdown of this supportive microenvironment can contribute to chronic disease will be discussed in this review. 

A range of conditions both acute and chronic are documented to modulate hematopoiesis. Signals that arise during infection, chronic disease, cancer, obesity, and ageing can elicit long-lasting changes to BM hematopoietic cells [40,41]. Often, these changes result in an imbalanced blood system typified by the overproduction of myeloid cells [14,21,41,42,43]. In the context of disease, for example, in diabetes or obesity, these hematopoietic changes contribute to the overall inflammatory burden and consequently play an active role in driving disease pathology [15,40,44,45]. The feedback of systemic inflammatory stimuli to the BM compartment likely perpetuates the chronicity of these inflammatory disorders by establishing a destructive myeloid-skewing bias [46,47,48,49,50]. As these metabolic-inflammatory conditions are increasingly prevalent in Western societies, more work is needed to fully understand the complex signals and regulatory networks that influence the differentiation decisions of BM progenitors. Great attention has recently been placed on the exogenous triggers capable of inducing innate immune memory or trained immunity in hematopoietic progenitors [51,52]. The non-specific “memory” that is induced by signals, such as β-glucan or the BCG vaccine, lead to long-lasting adaptations in BM progenitors and enhanced myelopoiesis. These findings show that the BM compartment can respond to a range of signals and that the demand for increased myeloid cells is not restricted to settings of infection or trained immunity but is also a major feature of the host response to chronic disease. In this review, we will highlight the ways in which the hematopoietic system and the supporting BM niche adapt in response to chronic disease and trained immunity. The underlying mechanisms that fine tune BM progenitor fate and function, including recent evidence for the contribution of the BM niche to disease pathogenesis, will be covered. 

## 2. Hematopoietic Adaptations in Chronic Inflammatory Disease

### 2.1. Diabetes-Mediated Changes to Hematopoiesis 

**Figure 1:**
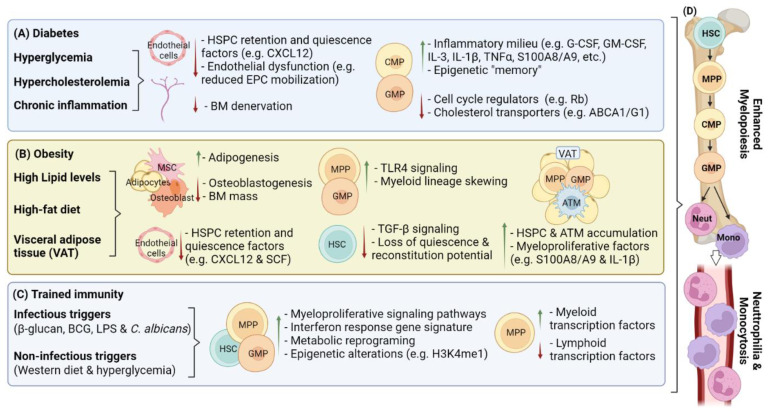
Hematopoietic adaptations in chronic inflammatory conditions. (**A**) Diabetes, (**B**) obesity, and signals capable of inducing (**C**) trained immunity dysregulate hematopoiesis and/or disrupt the BM niche. Activation of inflammatory signaling pathways, metabolic alterations and associated epigenetic changes combine to promote (**D**) myelopoiesis and the overproduction of monocytes and neutrophils. The oversupply of inflammatory cells is a factor driving the chronic nature of diabetes and obesity. In the context of trained immunity, increased myelopoiesis may be beneficial (e.g., host response to infection) or detrimental (e.g., chronic disease). ATM, adipose tissue macrophage; BM, bone marrow; CMP, common myeloid progenitor; GMP, granulocyte-macrophage progenitor; HSC, hematopoietic stem cell; HSPC, hematopoietic stem and progenitor cell; Mono, monocyte; MPP, multipotent progenitor; MSC, mesenchymal stromal cell; Neut, neutrophil; SNS, sympathetic nervous system; VAT, visceral adipose tissue. Created with BioRender.com (accessed on 31 January 2022).

Diabetes mellites (type 1 and type 2) is a metabolic disorder characterized by insulin resistance, impaired insulin secretion, hyperglycemia, dyslipidemia, and persistent inflammation [53]. Many of these diabetic complications have been shown to interfere with the normal hematopoietic function of the BM [54]. These diabetic-induced alterations to HSPCs and the BM niche often result in enhanced myelopoiesis and the overproduction of monocytes. The oversupply of inflammatory myeloid cells is often considered a factor driving the increased risk of developing cardiovascular disease in diabetes [55,56]. The BM compartment is now recognized as a key site in the pathogenesis of diabetes and its cardiovascular complications (Figure 1). 

Many studies have sought to address the effect of high glucose on the hematopoietic system. In a mouse model of diabetes, hyperglycemia promoted myelopoiesis by increasing the number and proliferation of CMP and GMP myeloid progenitors in the BM [57]. This led to elevated numbers of circulating neutrophils and Ly6C^hi^ monocytes in diabetic mice. Mechanistically, this study found that hyperglycemia-induced neutrophil production of S100 calcium-binding proteins A8/A9 (S100A8/A9) activated RAGE receptors on CMPs to drive myelopoiesis [57]. Reducing glucose levels in diabetic mice with sodium glucose co-transporter 2 inhibitor (SGLT2i) decreased S100A8/A9 levels in the plasma and led to reduced monocytosis and impaired monocyte entry into atherosclerotic lesions [57]. Multiple studies have implicated S100A8/9 and RAGE in diabetic inflammation and atherogenesis [58,59]. These mouse studies are consistent with data from patients with type 1 diabetes where a correlation between serum levels of S100A8/A9, monocytosis and the incidence of cardiovascular disease (CVD) has been identified [57,60,61]. The persistent risk of cardiovascular complications, even after lowering glucose, has been termed “hyperglycemic memory” [62]. A recent study found that high glucose levels promoted a proinflammatory macrophage phenotype [63], while hyperglycemia induced trained immunity in BM precursor cells and exacerbated atherosclerosis in transplantation studies [64], suggesting that high-glucose induces a form of “memory” in BM progenitor cells. These data fit with recent reports of BM progenitor reprogramming and the generation of a trained immune phenotype that leads to the production of myeloid cells with a heightened inflammatory potential [51,52]. The rewiring of the BM precursors by hyperglycemia may help explain the resistance of the complications of diabetes to conventional glucose-lowering therapies. 

Experimental evidence indicates that a major factor contributing to the development of CVD in diabetic patients is the interplay between hyperglycemia and hypercholesterolemia and their impact on hematopoiesis. Cellular cholesterol homeostasis is an important factor in the regulation of hematopoiesis, with increasing membrane cholesterol levels known to favor HSPC proliferation and a myeloid-lineage bias [50,65,66]. The cellular cholesterol content is partly regulated by cellular cholesterol efflux pathways that include the ATP-binding cassette (ABC) transporters, which drive cholesterol removal from the cell to efflux factors, such as apolipoproteins or lipoproteins [67]. These ABC transporters were originally implicated in governing macrophage cholesterol efflux [68] but have also been shown to play a critical role in the hematopoietic system [69,70]. The expression of the cholesterol transporters ABCA1 and ABCG1 on myeloid progenitors (i.e., GMP and CMP) in the BM is suppressed in a model of type 1 diabetes [71], while the levels of these transporters are reduced in circulating monocytes from diabetic patients [72,73,74]. The lack of ABCA1, ABCG1 [69], scavenger receptor type B-1 (SR-B1) [75], or the efflux factor apolipoprotein E (ApoE) [50] all result in intracellular cholesterol accumulation, enhanced HSPC proliferation and subsequent leukocytosis in the blood. Conversely, boosting the levels of efflux factors such as apolipoprotein A-I (ApoA-I) or high-density lipoprotein (HDL), which can unload cells of cholesterol in a transporter-independent manner, can restore normal HSPC proliferative responses [56,69]. These findings demonstrate the importance of cholesterol homeostasis to HSPC function. 

Cholesterol is a critical component of lipid rafts that form in the plasma membrane and act as signaling platforms for transmembrane receptors [76]. Changes in efflux pathways can dramatically affect raft formation and the signaling pathways of receptors contained within lipid rafts. Impaired efflux via ABCA1 and ABCG1 led to cholesterol accumulation in lipid rafts and increased expression of the β-subunit of the interleukin (IL)-3 and granulocyte–macrophage colony-stimulating factor (GM-CSF) receptor on the surface of HSPCs, leading to enhanced proliferative responses to these cytokines [50,69]. A high-fat diet was found to disrupt transforming growth factor (TGF)-β1 receptor signaling in lipid rafts, leading to downregulation of TGF-β1-induced HSC quiescence and elevated hematopoiesis [77]. MicroRNA 33 (miR33) is a transcriptional repressor of ABCA1 and ABCG1 in diabetes-associated hyperglycemic environments [74,78]. Rescue of ABCA1 expression with miR33 inhibitors restored cholesterol efflux and prevented hyperglycemia-induced monocytosis [71]. Hypercholesterolemia suppressed the expression of retinoblastoma protein (Rb), a cell cycle suppressor critical to HSPC quiescence, resulting in the expansion of HSPCs and a differentiation skew toward myeloid lineages [79]. The generation of reactive oxygen species (ROS) in response to hypercholesterolemia leads to HSPC expansion and loss of quiescence [80,81]. Impaired cholesterol efflux due to reduced HDL levels or dysfunctional HDL is observed in diabetic patients [82,83] and may contribute to the increased myelopoiesis in these patients. Aside from its influence on hematopoiesis in the BM, hypercholesterolemia also promotes stem cell mobilization and extramedullary myelopoiesis [66,84,85]. All told, these data support the notion that diabetes-associated leukocytosis is, in part, due to impaired cholesterol efflux within the BM niche, and therapeutic attempts to restore myelopoiesis to basal levels by boosting the availability of efflux factors warrant further investigation.

### 2.2. Diabetes-Mediated Changes to the Bone Marrow Niche

Diabetes disrupts the microenvironment of the BM niche, with the severity and duration of disease predicting the extent of the BM remodeling [86]. Diabetes leads to numerous abnormalities in the BM, such as small vessel disease (microangiopathy), nerve terminal damage (neuropathy), and impaired stem cell mobilization (mobilopathy) [87]. These pathologic manifestations are elicited by a dramatic shift in the local BM microenvironment, which includes changes to the inflammatory milieu and the cellular components of the niche (reviewed in [88]). Diabetes results in systemic dysfunction of endothelial cells [89], and there is diminished mobilization of endothelial progenitor cells (EPCs) from the BM into the blood in diabetes [90]. EPCs are a heterogenous population of cells derived from BM HSPCs [91] that play a central reparative/protective role in response to peripheral vascular damage [92]. Both type 1 and type 2 diabetics have a reduction in the number and function of circulating EPCs [93,94,95]. Reports from murine models confirmed the negative impact that diabetes has on EPC number and function (i.e., migration, network formation and increased vascular permeability) [96,97,98]. The poor release of EPCs from the BM in diabetes has been found to be due to defects in the local chemokine/growth factor networks and BM denervation [89,99,100,101]. Therapeutic efforts to boost EPC mobilization and function is considered a viable strategy to alleviate the vascular complications in diabetes [102]. A recent study found that endothelial cells in the BM niche of diabetic mice had diminished production of CXCL12, a HSPC retention and quiescence factor [103]. This detailed analysis across three diabetes models found that reduced BM endothelial CXCL12 production was accompanied by HSPC expansion and myelopoiesis. This study identified a novel epithelial growth factor receptor (Egfr) signaling pathway on BM endothelial cells that operates as an anti-inflammatory “brake” on diabetes-induced HSPC proliferation, myelopoiesis and wound repair [103]. Maintenance of vascular integrity is crucial for the physiologic function of the hematopoietic system; local depletion of HSPCs was observed in areas of increased oxidative stress and DNA damage [104]. Similar vascular changes have been found in human diabetes [86,105] and suggest that there may exist distinct zones within the niche that support HSPCs expansion and myelopoiesis [28,106]. Along these lines, higher rates of myelopoiesis were observed in close proximity to endothelial cells in a model of type 2 diabetes [103]. Vascular damage in diabetes is also driven by defects in the supporting and stabilizing pericytes whose function is diminished [96,107]. These studies underline the key role that BM endothelial cell function has on HSPC expansion, leukocyte production in diabetes, and its many complications. 

Chronic low-grade inflammation in diabetes has a deleterious impact on the BM niche and the resulting hematopoietic outcomes. Inflammatory cytokines, such as tumor necrosis factor (TNF), IL-1β, granulocyte colony-stimulating factor (G-CSF) and IL-3 are elevated in diabetic BM [98]. Hyperglycemia supports the production of TNF, a cytokine implicated in numerous diabetic complications, including damage to sensory nerves [108]. The sympathetic nervous system (SNS) is prominently involved in BM niche function [109] and BM SNS fibers are depleted in models of type 1 and type 2 diabetes [110]. Neuropathy is thought to precede diabetic complications, with the number of nerve endings inversely correlating with the number of HSPCs in the BM [99]. In diabetic models, BM denervation was shown to mediate many of the diabetic complications [111,112,113]. Damage to sensory nerves causes altered pain perception and impairs wound healing in diabetes. A recent study successfully employed gene therapy to restore BM innervation and function in a model of type 1 diabetes [114]. This type of approach enables cell-specific targeting in a complex multicellular organ. Apart from the overproduction of myeloid cells [54], disruption of the homeostatic functions of the BM niche also leads to increased release of so-called osteoprogenitor and smooth muscle progenitor cells in diabetic patients [115,116]. These populations arise from “differentiation drift” that is thought to be partly driven by hyperglycemia and inflammatory signals present in diabetes that favor the production of proinflammatory leukocytes [57,88,117]. Changes to the epigenetic profile of HSPCs also likely contribute to the lineage drift that leads to the persistent generation of myeloid cells with a proinflammatory phenotype in diabetes [118,119,120]. Understanding how the BM niche and HSPCs adapt to the many complex signals in diabetes will greatly advance our understanding of the condition and undoubtedly lead to new therapeutic opportunities. 

### 2.3. Obesity-Mediated Changes to Hematopoiesis 

Obesity is a first world epidemic, and with the accompanied low-grade inflammation, is thought to contribute to many co-morbidities, such as type 2 diabetes, CVD, and cancer development [121,122,123,124,125]. The inflammatory program in obesity impacts multiple organs and has many physiological consequences, including disruption of hematopoiesis, increased BM cellularity, lineage skewing and loss of BM integrity [45,49,126,127]. It is possible that the mechanisms driving hematopoietic disruption in obesity overlap, or are shared, with those observed in other obesity-related disease (e.g., diabetes and CVD). As such, understanding how the obesogenic environment impacts hematopoietic outcomes and how these changes link obesity to its many comorbidities is of considerable interest. The inflammatory signals and pathways that promote dysfunction within hematopoiesis and the wider BM microenvironment will be summarized (Figure 1). 

Adipose tissue expands in response to the elevated lipid levels present in obesity, which is followed by accumulation of immune cells, particularly adipose tissue macrophages (ATMs) [128]. Adipose tissue acts as an endocrine organ that produces numerous mediators that contribute to systemic inflammation and hematopoietic disruption [129]. Myelopoiesis is a key feature of obesity and drives the expansion of circulating monocytes and neutrophils and their eventual recruitment into the adipose tissue [130,131,132]. Understanding the inflammatory feedback loop between adipose tissue, BM and blood have been of great interest. Many studies over the previous decades have identified hematopoietic alterations in models of obesity [126,133,134,135,136]. Findings of a distinct bias toward myeloid cell expansion have been made in murine obesity models, exemplified by increased pools of myeloid progenitor populations, such as MPP3s, CMPs, GMPs and pre-granulocyte macrophages (pre-GMs) [49,135,137,138,139]. This myeloid bias is also present in obese humans where circulating monocytes and neutrophils are expanded and adopt a proinflammatory phenotype [140,141,142]. The obesity-associated impairment of lymphopoiesis is also thought to contribute to this myeloid–lymphoid imbalance [143,144]. The generation and activation of cell subsets, such as eosinophils, basophils, megakaryocytes, and thrombocytes are also impacted by obesity and its complications [145,146,147,148]. The influence of obesity on more primitive HSC populations is not as clear, with reports of the HSC pool increasing [49,137] and decreasing [135,136] in mouse models. HSCs mobilized to the spleen in obesity [135], while HSPCs were shown to accumulate in adipose tissue and sustain local ATM generation [149]. These peripheral HSC and HSPC populations may be key contributors to ATM accumulation in obesity, alongside the contributions made by the recruitment of BM-derived myeloid progenitors and local ATM proliferation [150]. Many reports have shown that macrophage depletion or inhibition is beneficial in obesity [151,152,153,154,155]. Therefore, the presence of functional HSPC populations in a variety of adipose tissue sites [156] raises important questions regarding their role in regulating the local macrophage population in obesity. The obesity-mediated alterations to HSPCs are persistent. Serial BM transplantation confirmed that HSPCs from obese mice had a sustained capacity to preferentially promote myeloid expansion and generate inflammatory ATMs [137]. A more recent murine study detailed that a short-term high-fat diet (HFD) of 4 weeks led to loss of quiescence and exhaustion of the most primitive HSCs. HFD disrupted TGF-β signaling on HSCs and reduced HSC number and function, an impact that could be reversed by treating mice with recombinant TGF-β [77]. Interestingly, 1-3 days of a HFD was sufficient to increase HSPCs and skew their differentiation toward the myeloid lineage [157]. These studies suggest that the hematopoietic alterations and myeloid skewing observed in obesity may not be due solely to the obesogenic environment. 

A role for visceral adipose tissue (VAT) in the myeloid bias in obesity was suggested from studies where VAT from leptin-deficient *Ob/Ob* mice was transplanted into wild-type recipients and recapitulated the obesity-associated myelopoiesis, neutrophilia and monocytosis [49]. Mechanistically, VAT from obese mice expressed higher levels of S100A8/A9, which as discussed earlier, is a major driver of hyperglycemia-induced monocytosis [57]. S100A8/A9-induced Toll-like receptor 4 (TLR4)/MyD88 and NLRP3-dependent production of IL-1β by resident ATMs [49]. This study suggests a positive feedback loop between ATM and myeloid progenitor populations in the BM with a key role for IL-1β. Relatedly, obesity-associated danger signals, such as free fatty acid, can be sensed by the NLRP3 inflammasome in both animals and humans to exacerbate obesity-induced inflammation and insulin resistance [158,159,160,161]. Studies have suggested the involvement of the TLR4/MyD88 axis in obesity-associated myelopoiesis [137,138], while also identifying a role for the TIR domain-containing adapter-inducing interferon-β (TRIF) adaptor [138]. Blockade of TLR4 signaling protects obese mice from loss of insulin sensitivity and excessive cytokine production [155] while also limiting fibrin deposition [162] and ATM activation [138,163]. TLR4 signaling was found to promote the transcription of genes involved in cell-cycle activity, myeloid activation, and differentiation in HSPCs isolated from obese mice [135]. Lipopolysaccharide (LPS) (and other microbial products) are thought to enter the circulation via the degradation of the gut barrier, leading to “leaky gut” [164]. LPS can rapidly reach the BM and activate TLR4 expressed on HSPCs [165]. Restoration of intestinal integrity improves metabolic and inflammatory parameters in obese mice [164]. The gut microbiota clearly impacts BM hemostasis [166]. The ability of a HFD to drive hematopoietic and BM niche alterations was mediated by alterations to the gut microbiota. Mice fed a HFD (versus those fed a normal diet), had poor HSC reconstitution, increased myelopoiesis and elevated BM adipogenesis at the expense of osteoblastogenesis [127]. Transplantation of stools from HFD to normal mice transferred these effects, whereas antibiotic treatment partially blocked the HFD-mediated effects on the BM niche. 

### 2.4. Obesity-Mediated Changes to the Bone Marrow Niche

There are a limited number of studies that directly explore how obesity-induced inflammation alters the cells that comprise the BM niche. However, it has been shown that bone architecture and the BM microenvironment is disrupted when an HFD is consumed. This is largely due to a change in the balance of mesenchymal stem cell (MSC) differentiation toward adipocytes and osteoblasts. An HFD-promoted MSC differentiation toward adipogenesis while impairing osteoblastogenesis, leading to reduced bone mass in mice [167,168]. The loss of bone mass and the associated skeletal changes driven by obesity in individuals [169], or in mice fed a HFD [167], were not due to impaired osteoclast activity. Increased adipogenesis in the BM occurs in situations, such as obesity [167,168], chemotherapy [170] and notably, dietary restriction [171,172]. The apparent paradox of the BM becoming enriched for adipocytes in obesity and dietary restriction suggests that BM adipogenesis may operate as a general response to systemic stress. The exact mechanistic role of BM adipocytes in regulating hematopoiesis is still unclear, but BM adipose tissue has been identified as a potent source of regulatory cytokines (e.g., CXCL12 and G-CSF) [170,173] and adipokines (e.g., adiponectin and leptin) [134,174,175] critical for retention, function, and maintenance of HSPCs. As discussed above, in a model of obesity-related diabetes, a role for BM endothelial cells in HSPC expansion and myelopoiesis was identified [103]. CXCL12, a well-known HSPC retention and quiescence factor [176], was downregulated in BM endothelial cells, resulting in higher HSPC activity [103]. Crosstalk between adipocytes and endothelial cells via secretion of extracellular vesicles enriched with bioactive molecules has been suggested as a novel form of communication between cells and tissues [177]. Both endothelial cells and perivascular stromal cells in the BM niche have been identified as sources of important hematopoietic mediators, such as stem cell factor (SCF) and CXCL12 [36,178,179,180]. SCF produced by leptin receptor (LepR+) cells in the BM was necessary to maintain the HSPC pool, including CMPs, GMPs and erythroid progenitors, whereas endothelial cell-derived SCF was essential for maintenance of HSCs [180]. This highlights the importance of discrete local BM microenvironments in the regulation of HSCs versus their more lineage-restricted progeny. 

Dietary restriction and physical exercise are two strategies that may limit or reverse some of the pathologic manifestations present in obesity. Voluntary physical activity (running) in mice reduced hematopoiesis and lowered circulating leukocyte levels. Physical activity reduced leptin release and signaling, leading to increased production in the BM of HSPC quiescence factors (e.g., CXCL12) by LepR+ stromal cells [181]. Voluntary exercise induced alterations in the HSPC epigenome that were maintained for 3 weeks and impaired the accessibility and expression of genes involved in proliferation and lineage fate without affecting emergency myelopoiesis. These findings are in stark contrast to the impact of involuntary or forced endurance training, which increased hematopoiesis resulting in leukocytosis [182,183]. Dietary intervention in obesity may also help restore BM hemostasis with reports of caloric restriction improving bone density and immunological memory [172,184]. A sedentary lifestyle and the associated low-grade inflammation present in obesity increase the risk of developing diabetes and CVD. Understanding how these factors and signals compromise hematopoiesis and the BM niche may help protect against obesity and its related complications. 

### 2.5. Hematopoietic Alterations in Trained Immunity

Trained immunity, a term proposed in 2011 [185], describes a long-lasting and broadly protective form of innate immunological memory. This unique immune adaptation can be generated in cells of the innate immune system by specific signals, enabling a program of heightened cellular responses to secondary challenge [186]. Epidemiological studies have noted that live attenuated vaccines can induce a state of trained immunity and that the cross-protective effects they enable can persist for months to years [186,187,188,189]. Recent studies have even suggested that trained immunity can be inherited in mammals mirroring observations made in invertebrates and plants [190,191,192]. The paradox of how this protection is sustained over such long periods, when the target innate immune cells are typically short lived (hours to days [193]), has only recently been resolved. Mitroulis et al. [52] found that HSPCs in the BM responded by extensively altering their metabolic and epigenetic profiles to enhance myelopoiesis and confer a protective secondary response. The discovery that trained immunity occurs at the precursor level underscores the central role that the hematopoietic system plays in shaping the innate immune system. A common feature of a trained immune state is the presence of a myeloid bias that is reminiscent of the emergency granulopoiesis or demand-adapted myelopoiesis that is often observed in response to infection, sterile inflammatory signals or in chronic disease [41,194,195] (Figure 1). The triggers of trained immunity include microbial stimuli such as the Bacillus Calmette–Guérin (BCG) vaccine [196], oral polio vaccine [197] and the measles vaccine [198]. The β-glucan the cell wall component of yeasts and fungi is used widely in studies of trained immunity [199], while the hepatitis B virus and the malarial pathogen *Plasmodium falciparum* were also found to build innate immune memory [200,201]. Many endogenous nonmicrobial signals have been shown to build innate memory such as oxidized low-density lipoprotein (LDL) [202], lipoprotein(a) [203], aldosterone [204] and uric acid [205]. All these triggers of trained immunity impact mature myeloid cell function with microbial signals typically having a more pronounced influence on the effector functions of the responding innate immune cells. We will outline the central signaling pathways and critical intermediates that build the different trained immunity programs below.

### 2.6. β-Glucan as a Modulator of Hematopoiesis and a Trigger of Trained Immunity

β-glucans are a group of heterogenous polysaccharides found in the cell walls of bacteria, fungi and yeast but are also highly enriched in barley, oats and seaweed [206]. β-glucan is one of the best-described drivers of trained immunity and is recognized by pathogen-recognition receptors (PRR), such as dectin-1 and complement receptor 3 that are expressed on innate immune cells [199,207,208,209]. In experimental models, β-glucan confers protection against chemotherapy-induced myeloablation, *Mycobacterium tuberculosis* (*Mtb*)*, Candida albicans* and *Leishmania* infection, while also boosting anti-tumor immunity [52,199,210,211,212,213,214]. The long-term protective effects induced by β-glucan are partly due to its ability to induce adaptations in HSCs that promote a myeloid bias in hematopoiesis. β-glucan administration induced the accumulation of the myeloproliferative mediators IL-1β and G-CSF in the BM [52]. This myeloid-skewing milieu led to the dramatic and sustained expansion of the myeloid-biased CD41^+^ LT-HSC and MPP3 subsets as well as increased GMPs. BM transplant studies confirmed that β-glucan induced a sustained increase in myelopoiesis [52]. This study showed for the first time that BM progenitor cells were reprogramed in the context of trained immunity and that these adaptations were essential for the long-term protective response. More recent in vivo studies have confirmed that β-glucan-mediated protection from secondary challenge is associated with the expansion of myelopoiesis [210,215]. IL-1β plays a central role in the hematopoietic changes driven by β-glucan, as disruption of the IL-1β signaling pathway impairs β-glucan-mediated myelopoiesis [52,210]. In another preclinical setting, β-glucan-mediated protection from tumor development was dependent on the type I interferon (IFN) signaling pathway in BM progenitors and neutrophils [211]. A role for type I IFN in BM reprograming has also been suggested in a Western-type diet (WD) model of immune training [48]. β-glucan-induced production of inflammatory mediators (e.g., IL-1β, G-CSF and type I IFN) facilitates profound changes in the cellular metabolic and transcriptional landscape that occur in hematopoietic cell populations. β-glucan increased the preference for glycolysis and boosted cholesterol synthesis in BM progenitor cells while also activating the GM-CSF signaling pathway [52]. These metabolic changes reflect those seen in mature myeloid cells [216,217]. Transcriptomic analysis of LT-HSCs from β-glucan treated mice revealed increased expression of cell-cycle genes and enrichment of innate immune pathway genes [52]. In vitro studies have highlighted a role for monomethylation of histone 3 lysine 4 (H3K4me1) in generating a trained immune phenotype [212,216,217,218]. H3K4me1 is associated with transcriptional activity [219], and the lysine methyltransferase Set7 is a key enzyme responsible for this modification [220]. The gene that encodes Set7 (i.e., *SETD7*) is increased in response to β-glucan [199], and Set7 activity was recently found to play a role in β-glucan-induced trained immunity, in particular, the expression of IL-1β and GM-CSF in the BM [221].

### 2.7. BCG Vaccine as a Modulator of Hematopoiesis and a Trigger of Trained Immunity

The BCG vaccine is widely used to induce protection in children against *Mtb* infection and is extensively studied in the context of trained immunity. Epidemiological and immunological studies have shown its ability to generate a long-lasting cross-protection against multiple pathogens, and even against cancer under experimental settings [196,222,223,224,225,226,227,228,229]. Intravenous administration of the BCG vaccine leads to its accumulation in the BM space, where it alters hematopoietic outcomes [51]. BCG administration led to the expansion of HSPCs, particularly ST-HSC and MPP subsets. Transcriptomic analysis revealed that myeloid-lineage transcription factors (e.g., *Cebpe*, *Cebpa*, and *Irf8*) were upregulated, while lymphoid-lineage transcription factors (e.g., *Pax5* and *Irf4*) were downregulated in BCG-exposed MPPs [51]. Consistent with this, flow cytometry data showed an increase in the number of myeloid-biased MPP3 subsets but not of the lymphoid-biased MPP4 subset in BCG-exposed mice [51]. These findings were validated in human volunteers where BCG vaccination led to persistent (up to 90 days) transcriptional and epigenetic alterations in HSPCs, which was accompanied by elevated myelopoiesis and enhanced innate immunity [230]. The transcriptional changes in HSPCs following BCG vaccination were found to relate to alterations in DNA accessibility in circulating monocytes [230]. BCG does not directly infect HSCs but may alter the local BM environment by infecting mature cells (e.g., macrophages) or by interacting with PRRs (e.g., NODs or TLRs) to induce inflammatory cytokine production [231,232]. The IFNγ signaling pathway was shown to play an indispensable role in the BCG-associated expansion of HSPCs, myelopoiesis and the protection against subsequent *Mtb* infection [51]. IFNγ controlled the epigenetic changes that support the expression of inflammatory mediators (e.g., IL-1β and TNF) in BCG-trained monocytes and macrophages [51]. A related study found that unlike BCG vaccination, *Mtb* infection impairs myelopoiesis by disrupting iron metabolism and inducing cell death in myeloid progenitors in a type I IFN-dependent manner [215]. This type I IFN/iron pathway enables *Mtb* immune evasion by inhibiting trained immunity and the activation of the innate immune system. This study also highlights the key role that the BM compartment plays in sustaining the effects of trained immunity, as the protective or detrimental signatures of BCG and *Mtb* on HSCs were found to be maintained for approximately one year [215].

Other infectious triggers of trained immunity have also been shown to alter hematopoiesis. LPS, a Gram-negative bacteria outer membrane component, generated trained immunity in HSPCs [233]. LPS-mediated training was associated with C/EBPβ-dependent chromatin alterations, which imparted epigenetic memory in HSCs that facilitated enhanced myelopoiesis (i.e., increased CD41^+^ LT-HSCs and GMPs) and protection from secondary *P. aeruginosa* infection [233]. Evidence for possible inheritance of the protective effects of trained immunity has recently been generated [190]. Trained immunity induced by sublethal infection with *C. albicans* or zymosan persisted across generations in mice. In the *C. albicans* model, offspring of *C. albicans*-exposed male mice were better able to respond and clear a subsequent challenge with *E. coli.* The increased resistance to *E. coli* infection was due to increased recruitment and activation of innate immune cells [190]. The protective effects were transgenerational, lasting from parents to F_2_ offspring. Mechanistically, hematopoietic rewiring toward increased production of “activated” myeloid cells was shown to support this immune phenomenon. The myeloid bias in the progeny of trained mice led to increased production of GMPs with transcriptional and epigenetic analyses revealing upregulation of genes involved in immune function and metabolism in intergenerationally trained myeloid progenitors [190]. Consistently, challenging offspring of zymosan A-exposed male mice with *Listeria monocytogenes* resulted in enhanced myelopoiesis and protection from infection [190]. The sperm of trained parental mice were found to have DNA methylation alterations at genes related to transcription factors involved in myelopoiesis. This particular finding raises the question of whether inheritance of a hematopoietic system skewed toward the overproduction of myeloid cells confers greater risk to the development of chronic disease in progeny (e.g., diabetes, obesity and CVD).

Non-infectious triggers also induce trained immunity by modulating HSPCs. A high-fat WD elevated levels of HSCs, MPPs and GMPs in mice, consistent with the production of trained immunity [48]. Transcriptional analysis revealed that genes upregulated in GMPs from WD-fed mice were associated with hematopoiesis, cell proliferation, immune activation, and a bias in GMP differentiation toward monocytic lineage [48]. More recently, hyperglycemia was shown to induce persistent reprogramming of BM HSCs, including promotion of glycolysis, increased myelopoiesis and generation of mature macrophages with a proatherosclerotic phenotype [64]. The discovery that BM progenitor cells adopt a form of “memory” in response to a range of training triggers helps explain how the effects of trained immunity can be maintained over long periods of time. What is currently unknown is whether cells of the BM niche contribute to the induction of trained immunity. Non-immune cells, such as endothelial cells and fibroblasts (both of which are major cellular constituents of the BM niche [234]), have been found to a adopt memory characteristics similar to that seen in innate cells [235,236]. Local production of developmental endothelial locus-1 (Del-1) [237] and G-CSF [39] by endothelial cells supports HSC expansion toward the myeloid lineage and endothelial cells express a range of PRRs that may enable interaction with various training stimuli [238]. Given that the BM niche plays an active role in the hematopoietic outcomes in chronic disease (as discussed above), it is likely that it also participates in the remodeling of hematopoiesis and the building of trained immunity.

## 3. Conclusions

The overproduction of immune cells is a major factor driving chronic disease. As these cells are often short-lived, the rate of their generation, recruitment, and accumulation within sites of disease shapes the inflammatory outcome in many conditions. Therefore, insights into the unique and common factors that elicit hematopoietic and BM niche adaptations in chronic disease and those that build trained immunity are of great importance.
